# An Emerging Role for RNA in a Memory-Like Behavioral Effect in *Aplysia*

**DOI:** 10.1523/ENEURO.0193-18.2018

**Published:** 2018-05-23

**Authors:** Rosalind SE Carney

**Highlighted Research Paper:** RNA from Trained *Aplysia* Can Induce an Epigenetic Engram for Long-Term Sensitization in Untrained *Aplysia*, by Alexis Bédécarrats, Shanping Chen, Kaycey Pearce, Diancai Cai and David L. Glanzman

For over a century, the predominant view in memory research has been that the engram, the physical substrate of memory, is stored at the synapse. Research from several laboratories has identified roles for protein synthesis and methylation during critical time periods of memory consolidation and storage (for review, see Day and Sweatt, 2010; Kandel et al., 2014; Marshall and Bredy, 2016; Poo et al., 2016). However, research in the sea snail, *Aplysia*, suggests that although protein synthesis and methylation remain universal to the consolidation of long-term memories in this species, the cellular location in which memory storage occurs may differ. David Glanzman (UCLA) and his laboratory have exploited the simplicity of the *Aplysia* nervous system for memory research because of several unique features of this model. Their research implicates the nucleus as a cellular location of memory storage in *Aplysia* (Pearce et al., 2017).

**Figure 1.**
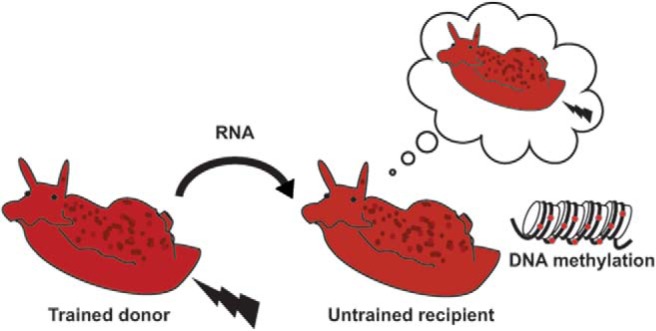
*Aplysia* were trained with electrical shocks delivered to the tail. The training produced long-term (>24 h) sensitization of the snail defensive withdrawal reflex. RNA was then extracted from the nervous system of the trained snails and injected into untrained, naive, snails. The defensive withdrawal reflex was enhanced in the naive recipients. Other snails that received injections of RNA from untrained donor snails did not subsequently exhibit enhancement of the reflex. Additional evidence from the study supports the idea that the RNA from the trained donors induced memory-like changes in the behavior of the recipients via an epigenetic change, DNA methylation. (Image courtesy of David Glanzman, UCLA.).

*Aplysia* only has 20,000 neurons, many of which are mapped and understood in terms of function, circuitry, and behavior. Long-term sensitization of a simple defensive behavior, called the siphon withdrawal reflex (SWR), can be taught by repeated electrical tail shocks during training (Fig. 1). The training results in a form of nonassociative learning, long-term sensitization, in *Aplysia*, meaning that the animals more strongly execute the SWR when a subsequent touch is applied to the siphon during testing.

In their *eNeuro* publication, Bédécarrats et al. (2018) further their alternative hypothesis of memory storage in the nucleus with four main findings:

1) RNA extracted from the CNS of sensitized (trained) donor *Aplysia* induced behavioral sensitization when injected into the CNS of untrained *Aplysia* recipients.
2) The behavioral effect of the RNA-induced sensitization in the untrained recipients required DNA methylation.
3) Sensory neuron hyperexcitability, a cellular mechanism of long-term sensitization, was replicated *in vitro* by exposing sensory neurons dissociated from untrained *Aplysia* to RNA isolated from trained *Aplysia*.
4) The *in vitro* effect of RNA isolated from trained *Aplysia* was specific to sensory neurons, but not to motor neurons, dissociated from untrained *Aplysia*.

It is important to emphasize that these findings do not suggest that memory is encoded by RNA. Rather, they indicate that RNA is sufficient to generate a priming component of the engram for long-term sensitization, and that RNA transfer appears to induce epigenetic, nonsynaptic changes that mediate a behavioral effect in *Aplysia* that resembles the effect produced by the electrical shocks in trained animals.

From these findings, many interesting questions remain. What is the identity of the particular species of donor RNA that primed the behavioral and cellular changes in the untrained recipients? Also, it will be important to identify the changes in expression of the methylated recipient genes at the time the behavioral modification occurs. Bédécarrats et al. (2018) also acknowledge that the cellular changes they observed *in vitro* are unlikely to fully account for the behavioral changes, as interneuronal circuits also regulate the SWR.

Why is the engram so important? Knowing the neural substrate of memory is crucial for therapeutic applications of memory manipulation, which can have opposing aims. For example, someone with Alzheimer’s disease has difficulty remembering and maintaining memories, whereas someone with post-traumatic stress disorder can have strong memories of disturbing events. It is unknown if the current findings will be relevant to species other than *Aplysia*. If Glanzman’s hypothesis that RNA could impact in memory storage in humans is confirmed, this could represent a tremendous potential for treatments to counter memory loss or enhance memory suppression. In the scenario of potential human therapies, it may be beneficial to directly target the methylation process as epigenetic modifications have long-lasting effects.

The concept of memory storage in the nucleus is highly controversial to the long-standing, dominant hypothesis of synaptic memory storage. If replicated in mammals, could it represent an additive mechanism of memory storage? As yet, no one knows, but this is undoubtedly an exciting and dynamic topic in neuroscience to watch.
